# Prion in Saliva of Bovine Spongiform Encephalopathy–Infected Cattle

**DOI:** 10.3201/1812.120528

**Published:** 2012-12

**Authors:** Hiroyuki Okada, Yuichi Murayama, Noriko Shimozaki, Miyako Yoshioka, Kentaro Masujin, Morikazu Imamura, Yoshifumi Iwamaru, Yuichi Matsuura, Kohtaro Miyazawa, Shigeo Fukuda, Takashi Yokoyama, Shirou Mohri

**Affiliations:** Author affiliations: National Agriculture and Food Research Organization, Tsukuba, Japan (H. Okada, Y. Murayama, N. Shimozaki, M. Yoshioka, K. Masujin, M. Imamura, Y. Iwamaru, Y. Matsuura, K. Miyazawa, T. Yokoyama, S. Mohri);; Hokkaido Research Organization, Shintoku, Japan (S. Fukuda)

**Keywords:** antemortem diagnosis, bovine spongiform encephalopathy, BSE, prion protein, prion, protein misfolding cyclic amplification, saliva, cattle, prions and related diseases

**To the Editor:** A definitive diagnosis of bovine spongiform encephalopathy (BSE) in cattle usually relies on Western blot and immunohistochemical testing of samples from the obex region of the brainstem. These conventional diagnostic tests can detect the presence of the abnormal (disease-associated) form of the prion protein (PrP^Sc^) in brain samples several months before the onset of clinical signs; however, there is no appropriate, universal tool for early preclinical and antemortem diagnosis of BSE. Furthermore, confirmation of the disease is currently only possible by postmortem examination of brain tissues. In this study, we used the serial protein misfolding cyclic amplification (sPMCA) technique to determine the presence of PrP^Sc^ in saliva samples collected from BSE-infected cows before and after the onset of disease (*1*).

In a previous study (*2*), we analyzed the tissue distribution of PrP^Sc^ in cattle up to 66 months after they were orally inoculated with a relatively low dose (5 g) of homogenized brainstem from animals with naturally occurring BSE in England. In 2011, after publication of that study and 83.3 months after the cows were inoculated, clinical signs of BSE developed in 1 cow (no. 5444); necropsy was performed 84.7 months after inoculation. In addition, we used saliva samples from 2 BSE-affected cows (nos. 5413 and 5437) (*2*) to determine the presence of PrP^Sc^.

We collected saliva samples from animals at 4 monthly intervals, beginning in 2009, 56 months after inoculation. Samples were stored at −80°C until analysis. Using the sodium phosphotungstic acid precipitation method, we concentrated (100-fold) individual 1-mL saliva samples from each time point. We then diluted the concentrated samples 1:10 with the normal isoform of prion protein substrate containing 0.5% potassium dextran sulfate. Using the sPMCA technique as described (*1*), we amplified the samples in 3–8 tubes, and we used Western blot to analyze the proteinase K–treated sPMCA products (*2*).

Using Western blot and immunohistochemical tests, we detected the accumulation of PrP^Sc^ in brains collected at necropsy from the 3 cows examined. In addition, using the sPMCA technique, we detected PrP^Sc^ signal in 1) saliva samples that were concentrated from samples collected from the same 3 cows at necropsy and in 2) concentrated saliva samples that were collected from 2 of the cows (nos. 5413 and 5444) at the early clinical stages of disease. 

After saliva samples underwent 3 rounds of amplification, we detected PrP^Sc^ in a saliva sample that was collected from cow number 5437 two months before the clinical onset of clinical symptoms (Figure). For 2 of the cows (nos. 5413 and 5437), the positive ratio of salivary PrP^Sc^ at round 4 of amplification increased as the disease progressed (Figure). Because PrP^Sc^ signal could be detected in BSE-infected brain homogenates diluted up to 10^−10^ after 2 rounds of amplification (*1*), we estimated PrP^Sc^ levels in the nonconcentrated original saliva samples to be lower than those in BSE-infected brain homogenate diluted to 10^−12^. No PrP^Sc^ signal was detected in samples collected from the 3 cows 3–5 months before the onset of clinical symptoms or from age-matched noninfected controls, even after 4 rounds of amplification.

**Figure:**
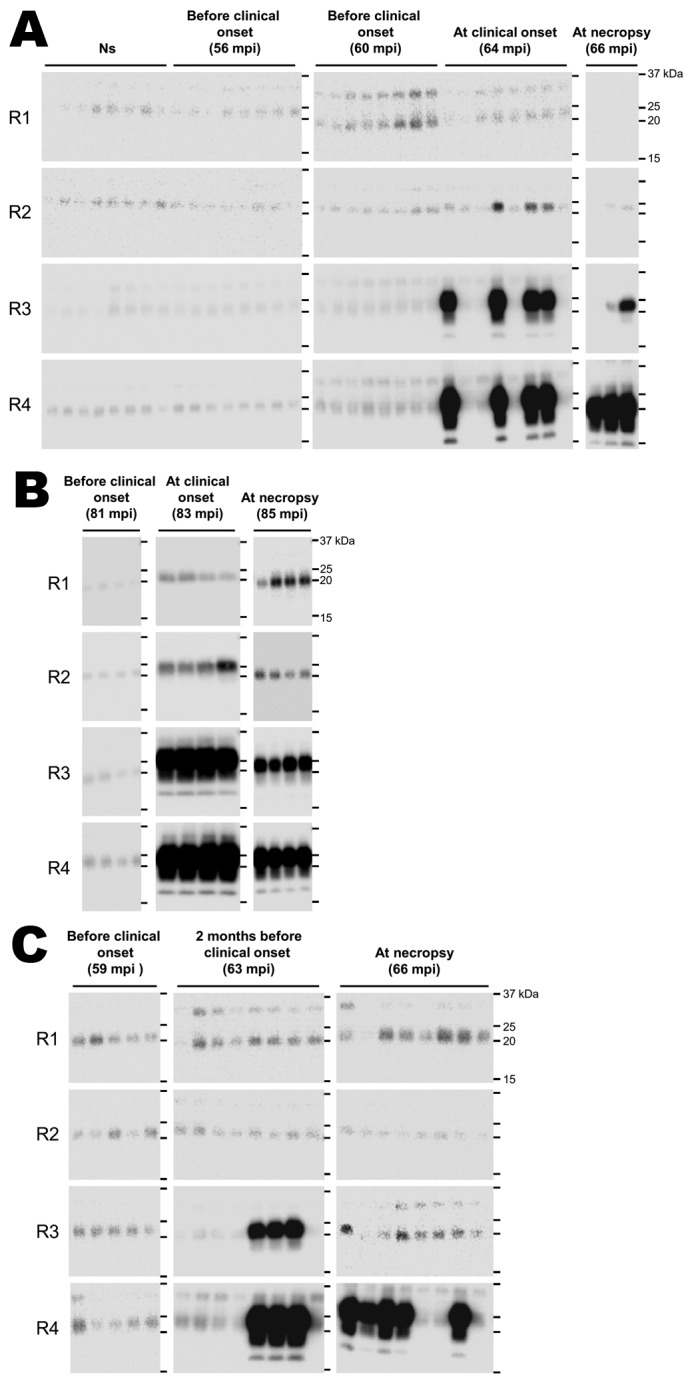
Western blot detection, using the serial protein misfolding cyclic amplification technique, of the abnormal (disease-associated) form of the prion protein (PrP^Sc^) in concentrated saliva samples from 3 cows experimentally infected by inoculation with the agent of bovine spongiform encephalopathy: cows 5413 (A), 5444 (B), and 5437 (C). PrP^Sc^ was detected in saliva samples at the initial clinical and terminal stages of the disease (A, B). PrP^Sc^ was also detected in a saliva sample, after 3 rounds of amplification, obtained 2 months before the onset of clinical symptoms in 1 of the 3 cows (C). All saliva samples were concentrated by using the sodium phosphotungstic acid precipitation method. After protein misfolding cyclic amplification, extra bands with a molecular weight higher than that for PrP^Sc^ were occasionally observed, likely corresponding to prion protein aggregates or to residue of the normal isoform of prion protein resulting from incomplete proteinase K digestion. Molecular mass markers (in kDa) are shown on the right. R1–R4, rounds 1–4 of amplification; Ns, no seed control; mpi, months postinoculation.

We demonstrated the presence of PrP^Sc^ in saliva of BSE-affected cows during the clinical stage of the disease, and in 1 case, at the preclinical or asymptomatic stage. Our findings suggest that PrP^Sc^ is likely to be detected in the saliva of BSE-affected cattle during the clinical stage of disease, after accumulation of PrP^Sc^ in the brain. PrP^Sc^ was found in the salivary glands of BSE-affected cattle at the terminal stage of infection (*1*). Therefore, once the infectious agent reaches the central nervous system, it may spread centrifugally from the brain to the salivary glands through the autonomic nervous system.

Infectivity of saliva and the presence of PrP^Sc^ in saliva have been reported in other ruminants affected with transmissible spongiform encephalopathy. Infectivity of saliva was demonstrated in deer with chronic wasting disease (*3*) and in scrapie-affected sheep (*4*); the immunolabeled PrP^Sc^ accumulated in the salivary glands of scrapie-affected sheep (*5*). A low level of PrP^Sc^ was detected in concentrated buccal swab samples of preclinical scrapie-infected sheep by using sPMCA (*6*,*7*). These results suggest that small amounts of PrP^Sc^ may accumulate in the salivary glands and are then secreted into saliva.

The presence of infectious prions in saliva may explain the facile horizontal transmission of scrapie in sheep (*4*–*6*) and chronic wasting disease in deer (*4*,*8*). There has been no epidemiologic evidence, however, that saliva, milk, blood, and cerebrospinal fluid from BSE-infected cattle are infectious (*9*). Nonetheless, the potential risk for BSE transmission by body fluids or excretions from BSE-infected cattle is cannot be ruled out by the current data.
